# Acid tolerance properties of dental biofilms in vivo

**DOI:** 10.1186/s12866-017-1074-7

**Published:** 2017-07-25

**Authors:** A Senneby, JR Davies, G Svensäter, J Neilands

**Affiliations:** 1 Department of Oral and Maxillofacial Radiology, Malmö University, Faculty of Odontology, 205 06 Malmö, SE Sweden; 2 Department of Oral Biology, Faculty of Odontology, Malmö University, Malmö, Sweden

**Keywords:** Microbiology, Phenotype, Plaque, pH, Observer performance, Acid tolerance

## Abstract

**Background:**

The ecological plaque hypothesis explains caries development as the result of the enrichment of acid tolerant bacteria in dental biofilms in response to prolonged periods of low pH. Acid production by an acid tolerant microflora causes demineralisation of tooth enamel and thus, individuals with a greater proportion of acid tolerant bacteria would be expected to be more prone to caries development. Biofilm acid tolerance could therefore be a possible biomarker for caries prediction. However, little is known about the stability of biofilm acid tolerance over time in vivo or the distribution throughout the oral cavity. Therefore the aim of this study was **t**o assess intra-individual differences in biofilm acid-tolerance between different tooth surfaces and inter-individual variation as well as stability of acid tolerance over time.

**Results:**

The majority of the adolescents showed low scores for biofilm acid tolerance. In 14 of 20 individuals no differences were seen between the three tooth sites examined. In the remaining six, acid-tolerance at the premolar site differed from one of the other sites. At 51 of 60 tooth sites, acid-tolerance at baseline was unchanged after 1 month. However, acid tolerance values changed over a 1-year period in 50% of the individuals.

**Conclusions:**

Biofilm acid tolerance showed short-term stability and low variation between different sites in the same individual suggesting that the acid tolerance could be a promising biological biomarker candidate for caries prediction. Further evaluation is however needed and prospective clinical trials are called for to evaluate the diagnostic accuracy.

## Background

The oral microbiota plays an important role in health by preventing colonisation of the oral cavity by pathogenic species [1]. The human oral cavity exhibits a high degree of bacterial diversity and more than 700 taxa have been identified in meta-genomic studies [2]. Bacteria grow in complex multi-species biofilms on the hard and soft-oral tissue surfaces with saliva or gingival exudate as the major nutrient sources. Biofilms on the teeth (dental plaque) above the gingival margin are dominated by sacharolytic bacteria, which generate energy by breakdown of carbohydrates from salivary glycoproteins as well as ingested food, through the glycolytic pathway. Pyruvate conversion then results in acidic end-products, including lactic acid, which can rapidly lower the pH in dental biofilms. If the pH remains lower than 5.5 for a prolonged period, demineralisation of the enamel will occur [3, 4].

As well as causing demineralisation, acids generated from carbohydrate metabolism by members of the biofilm affect the ecology of the biofilm itself. Prolonged periods of low pH in biofilms favour growth of intrinsically acid-tolerant (aciduric) bacteria such as Lactobacilli and Bifidobacteria, leading to increased proportions of these species [5]. Experiments using the model bacterium *Streptococcus mutans* have shown that, although not intrinsically aciduric, oral streptococci can survive acid stress through the induction of an acid-tolerance response (ATR) when exposed to a sub-lethal pH (~ pH 5) [6, 7]. Acid tolerance is the ability of a bacterium to sense and respond to acid stress [8] and is a phenomenon first discovered in *Salmonella enterica* serovar *typhimurium* [9]. Later it has been shown to occur in several other species including *Escherichia coli*, *Listeria monocytogenes*, *Bifidobacterium longum* and oral bacteria such as *Enterococcus hirae*, *Streptococcus gordonii*, *Streptococcus sanguinis* and *Lactobacillus casei* [7, 10–13]. The ATR in *S. mutans* involves, for example, changes in cell membrane composition, exclusion and extrusion of protons from the cytoplasm, generation of alkali from arginine-containing proteins and up-regulation of chaperones and nucleases to prevent protein misfolding and maintain DNA integrity respectively. [6, 14–18]. In addition to these survival mechanisms, a shift in the pH optimum for glucose transport and glycolysis allows acid-tolerant streptococcal cells to continue metabolising carbohydrates and producing acid end-products even in acidic environments [6]. Thus, frequent exposure to low pH will favour, not only intrinsically aciduric species, but also bacteria capable of eliciting an ATR [1, 19]. Selection of intrinsically and adapted acid-tolerant bacteria eventually results in a highly aciduric plaque that promotes demineralization of the enamel [20] and development of caries lesions [1, 21–24]. Consequently, individuals with highly aciduric plaque would be expected to have an increased risk of developing caries compared to those within non-aciduric biofilms.

Caries is one of the most common biofilm-mediated diseases in the world and around 10% of the population worldwide suffer from severe disease. In order to target preventative measures most effectively, it is essential to be able to identify those individuals with increased risk of developing severe caries. Traditionally, methods for caries risk assessment include previous caries experience, sociodemographic- or socio-economic factors, salivary secretion rate and buffer capacity as well as oral hygiene parameters [25–27]. In addition, microbiological tests that measure the number of mutans streptococci or lactobacilli in saliva or plaque are used. These tests show high specificity but low and scattered sensitivity [25–28]. Thus, individuals who are not at risk of developing caries are effectively identified whereas those with increased risk are not. There are several possible reasons for the low sensitivity seen for tests that rely on the count of specific bacterial species, (e.g. *S. mutans*). For example, it is well known that dental plaque harbours other streptococci that are capable of exhibiting an acid tolerant phenotype (e.g. *S. gordonii* and *Streptococcus oralis*) that would lead to a false negative result with this kind of test [20]. On the contrary, the presence of non-aciduric *S. mutans* in the sample would give rise to false positive results. The low sensitivity of existing tests for caries prediction, clearly illustrates a need for new approaches.

Attempts have been made to develop methods that detect the actual level of acid-tolerance in dental plaque. These include enumeration of bacteria after culturing on low pH agar, where only aciduric bacteria can grow [29] and measurement of growth in acidified broth in microtiter plates [30]. Another method of distinguishing acid-tolerant bacteria from non-acid-tolerant ones is by exposing an established biofilm (dental plaque sample) to an acid challenge (i.e. a pH known to kill non-acid-tolerant bacteria) and visually assessing the proportion of bacteria that survive by staining with the LIVE/DEAD® BacLight™ stain [31]. The latter method has been used previously to determine the levels of acid-tolerant bacteria in dental plaque samples from exposed root surfaces in elderly patients [32]. Since the presence of acid tolerant microorganisms in biofilms is intimately related to the demineralisation process of the tooth, the acid challenge method could be a possible test in caries prediction. However in order to be a possible candidate it must not fluctuate over the short term and preferably show low variation within the oral cavity so that sampling can be easy and representative. In this study we have used the acid challenge method to investigate biofilm acid tolerance at different tooth sites in the same individual as well as the variation between individuals. In addition, short and long-term changes in acid tolerance have been studied. This work represents the first step in the evaluation process of biofilm acid tolerance in vivo as a possible biomarker in caries prediction.

## Methods

### Subjects

Forty adolescents (aged 12–13 years) visiting a public dental health clinic in Kronoberg County, Sweden for their regular dental check-up appointment were recruited to the study. Ethical approval was obtained from the Regional Ethical Review Board, Lund, Sweden (registration number: 2016/146). Adolescents and parents received written information by post prior to the visit and informed consent was obtained before enrollment in the study. Eligible subjects were healthy individuals with erupted permanent first molars, first premolars and incisors. Individuals who had received antibiotic treatment over the past 3 months were excluded.

### Sample collection

Plaque biofilm sampling was performed by a dental hygienist, who was trained in the procedure. For half of the adolescents (*n* = 20) biofilms were sampled using Quicksticks (Dab Dental AB, Upplands Väsby, Sweden) from all supragingival approximal surfaces between second premolars and first molars (four sites) and pooled to give one sample for each individual. Samples were taken at baseline (time 0), after 6 and 12 months. In the other individuals (*n* = 20) biofilms were sampled and pooled in the same manner from all approximal surfaces between second premolars and first molars (four sites), between canines and first premolars (four sites) and between the central incisors in both jaws (two sites) resulting in three samples from each individual. This sampling took place at baseline, after 3 days and after 1 month. Samples were transferred to sterile microfuge tubes and sent to the laboratory for analysis.

### Assessment of biofilm acid tolerance

**Fig. 1:**
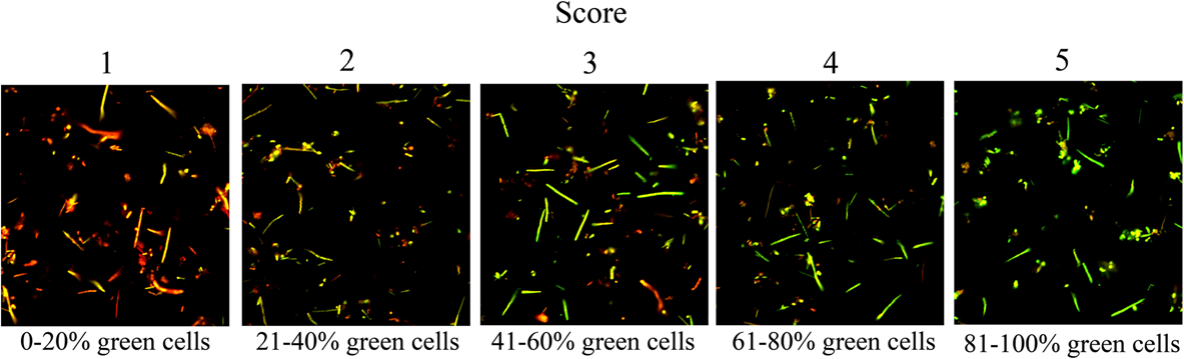
Images representing the different scores. Acid tolerant bacteria appear *green* and non-acid tolerant bacteria *red* when being stained with LIVE/DEAD® BacLight™ Viability stain

Each sample was suspended in 200 μl TYE medium (1.7% tryptone, 0.3% yeast) containing 40 mM phosphate/citrate (P/C) buffer (pH 3.5) and 20 mM glucose and vortexed with glass beads to disperse the biofilm before incubation at 37^°^C for 2 h. Cells were then stained using LIVE/DEAD® BacLight™ Fluorescent Stain (Molecular Probes, Eugene, USA) [32] and introduced into Ibidi mini flow cells (IbidiR μ-Slide, Ibidi GmbH, Martinsried, Germany). Each sample was examined using an inverted confocal scanning laser microscope (Nikon Eclipse TE2000, Nikon Corp., Tokyo, Japan) with an Ar laser (488 nm laser excitation). Live (acid-tolerant) bacteria appeared green while dead (non acid-tolerant) bacteria appeared red (Fig. 1). Assessment of acid-tolerance was based on scoring of ten random images from each sample. Due to differences in bacterial size and cellular morphology, it was not possible to estimate the percentage of green (acid-tolerant) and red (non-acid-tolerant) bacteria using pixel-based methods. Assessment was therefore carried out by an experienced rater (author JN) through comparison of the proportion of green cells in each image with an interval scale (with 5 possible scores or threshold values) presented in Fig. 1 [33]. A change in the level of acid-tolerance was defined as an increase or decrease of ≥2 scores according to the interval scale. This corresponded to a difference of at least 20% in the proportion of acid-tolerant cells in the images. Scoring is more time efficient than counting the cells in each image manually, especially when a large number of images are to be analysed. To test the validity of the interval scale (1–5 scores) for assessment of the proportion of acid-tolerant cells in biofilms, a comparison was made of the score given by the rater with that given by counting the actual numbers of acid-tolerant cells in 10 images (2 images representing each score). The intra-rater agreement of acid-tolerance assessments according to the interval scale was determined by comparing the scores given for the same 50 images, presented in random order, on two separate occasions separated by 2 weeks. The 50 images represented 10 images for each score.

### Statistical analysis

Wilcoxon’s matched-pairs signed rank test was used for the comparison of score values from baseline with the corresponding values from day 3 and 1 month. The score values from the different sites at baseline as well as the score values from baseline, 6 months and 1 year were compared with a 1-way ANOVA (Friedman’s test with Dunn’s multiple comparison post-test).

## Results

### Validity of interval scale for assessment of acid-tolerance in biofilms

Acid tolerance in this study was defined as the proportion of biofilm cells that were green (i.e. viable following an acid challenge (pH 3.5) for 2 h) by visual comparison with the scale shown in Fig. 1. For eight images, the score assigned using the scale was exactly the same as that revealed by counting. For two images however, the rater’s assessment differed by one score from that obtained by counting. In both images, the actual number of acid-tolerant cells was close to the threshold boundary, for example, an image given score 3 by the rater (corresponding to 41–60% acid-tolerant cells) actually contained 62% acid-tolerant cells.

The intra-rater agreement was 94% (kappa 0.92) as the exact same score was given to 47 images on the two occasions. In the remaining 3 images, the results differed by one score (defined as no difference). Thus, the intra-rater agreement according to Landis and Koch was almost perfect [34].

### Distribution of acid-tolerance in 12-year old individuals

**Fig. 2:**
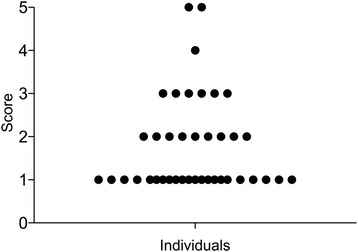
Distribution of acid tolerance scores at baseline. Each dot represents one individual in the study

**Fig. 3:**
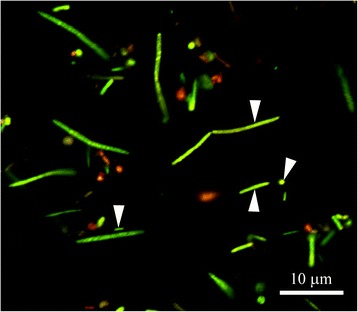
Image of biofilm sample showing acid tolerant bacteria (*green cells*) with different morphologies

To determine the range of acid-tolerance scores within the study population, biofilm samples were taken on a single occasion from the approximal surfaces between the second pre-molar and first molar in each quadrant and pooled to give one sample from each individual. This revealed a spread in biofilm acid-tolerance amongst the 40 individuals over the entire range of the evaluation scale (from score 1, 0–20% to score 5, 81–100%). Most of the individuals (75%) had a low acid-tolerance (score 1 or 2) while less than 10% had biofilms with a high acid-tolerance (score 4 or 5) (Fig. 2). Thus, the level of acid tolerance within the study population varied, with the majority of individuals showing low levels of acid-tolerance in their oral biofilms. In images of biofilms from the individuals showing a high acid tolerance, both green cocci and short, long and pleomorphic, green rods could be observed, suggesting that a range of different bacterial species in the biofilms were acid-tolerant (Fig. 3).

### Comparison of acid tolerance between different tooth surfaces in the same individual

To investigate whether acid-tolerance varied between tooth surfaces in the same individual, biofilms were sampled on a single occasion from three different approximal sites in 20 subjects. In all individuals, the sample taken from between the first incisors and that taken between the second premolar and the first molar showed exactly the same acid-tolerance score. In 14 ﻿subjects, the approximal site between the canines and the first premolar had the same acid-tolerance score as the incisor and molar sites. However, in the remaining six individuals, the acid-tolerance at the pre-molar site differed from one of the other sites by ≥2 scores (*p* = 0.17) (Table 1).

**Table 1:** Distribution of acid tolerance scores in biofilms in individuals exhibiting a change in score (change = difference in score ≥ 2) between the three different sites within the oral cavity (between the central incisors, approximal surface between canine and of 1st premolar and approximal surface between 2nd premolar and 1st molar). Biofilms collected from all quadrants was pooled in one sample

Individual	Inscisors	1st premolars	1st molars
A.	1	3	1
B.	3	1	2
C.	2	1	3
D.	2	1	3
E.	3	1	2
F.	2	1	3

### Reliability of the acid tolerance assessment method

To investigate the reliability of the assessment method, sampling from between the central incisors, canine and first premolar, and second premolar and first molar sites was repeated after 3 days and the acid-tolerance values compared with the baseline measurements. This revealed a high degree of short-term consistency, with the same values obtained in 18 of the 20 individuals at the incisor and molar sites and 17 of 20 the individuals at the sites between the canines and pre-molars (*p* = 0.48).

### Changes in biofilm acid-tolerance over time

To investigate the time-frame over which changes in biofilm acid-tolerance occurred, the values at baseline at 60 sites were compared with those obtained after 1 month. At 51 of 60 sites, low (score 1 and 2) or moderate (score 3) acid-tolerance values at baseline were unchanged after 1 month (Table 2). At four sites an increase (≥ 2 scores) in acid-tolerance was seen, whereas at five sites a decrease (≥ 2 scores) was seen. These data suggest that the proportion of acid-tolerant organisms in biofilms can both increase and decrease over 1 month but in the majority of the sites it remains unchanged (*p* = 0.63).

**Table 2:** Change in biofilm acid tolerance scores (change = difference in score ≥ 2) between baseline and 1-month follow-up in 60 tooth sites (biofilm collected from 20 individuals, three locations; between central inscisors, approximal surface between canines and 1st premolar and approximal surface between 2nd premolar and 1st molar. Biofilm collected from all quadrants and pooled in one sample)

No change in acid tolerance score		Increased acid tolerance score		Decreased acid tolerance score	
Baseline – 1month^a^	Sites (*n*)	Baseline – 1month^a^	Sites (*n*)	Baseline – 1month^a^	Sites (*n*)
1–1	26	1–4	2	3–1	4
1–2	9	1–3	1	4–1	1
2–1	6	2–4	1		
2–2	1				
2–3	3				
3–2	2				
3–3	2				
Total (*n*):	51		4		5

^a^ *Acid tolerance score*

*n = number*

Since we postulate that caries development would require a high-degree of acid tolerance in the biofilm over a considerable period, fluctuation in biofilm acid-tolerance at one site over the longer term (6 months to 1 year) was also studied. Eighty-five percent of the sites showed a low or moderate acid-tolerance at baseline and the values fluctuated around this level over the one-year period (Table 3). At two sites however, a high acid-tolerance measurement at baseline (score 5) decreased to a low value at 6 months (score 1), which was maintained at the one-year sampling point. In contrast, at 1 site the opposite occurred with a low initial acid tolerance score increasing to a high score after 6 months that then remained stable at 1 year. These results show that for most individuals in this study, acid tolerance is generally low and stable over the long-term (*p* = 0.88). However, a small proportion of the individuals showed changes between the values obtained on the first and second sampling occasions and that once a change had occurred, the new level of acid tolerance was maintained at the one-year sampling point.

**Table 3:** Distribution of acid tolerance scores in biofilm collected from approximal surfaces between 2nd premolars and first molars from 20 individuals at baseline, 6-months and 1-year follow-up

Individuals	Baseline	6 months	1 year
1.	1	1	1
2.	1	1	1
3.	1	1	1
4.	1	1	2
5.	1	2	1
6.	1	2	1
7.	1	3	1
8.	1	3	2
9.	1	3	3
10.	1	5	4
11.	2	1	2
12.	2	1	2
13.	2	2	2
14.	2	3	1
15.	3	1	2
16.	3	2	1
17.	3	2	2
18.	3	2	4
19.	5	1	1
20.	5	1	1

## Discussion

Supragingival plaque biofilms develop through adherence of microorganisms to saliva-coated tooth surfaces. These biofilms show a high degree of diversity but are dominated by *Streptococcus* and *Actinomyces*, which express adhesins that can bind adsorbed salivary proteins. Both these genera are saccharolytic and normally acquire nutrients through the cooperative degradation of glycan chains from salivary glycoproteins. Intermittently carbohydrates derived from food are metabolized by microorganisms, giving rise to products such as acetate, lactate, formate and succinate, which lead to rapid acidification of the biofilm [3]. As early as 1944, Stephan [33] showed that the pH in plaque from caries-active individuals was initially lower and fell to a lower level for a longer period in response to a glucose rinse, than in plaque from healthy subjects. The phenomenon has been confirmed in subsequent studies; see for instance Aranibar Quiroz et al. [35], where, in caries-active individuals, biofilm pH remained below 5.5 for more than 15 min after a sucrose rinse. This observation has been interpreted as evidence that biofilms showing sustained pH drops contain acid-tolerant bacteria, which survive the long-term acidic environment and are able to metabolize and generate acid at low pH levels [36]. In addition to adapted acid-tolerant species such as members of the genera *Streptococcus* and *Actinomyces* acid tolerant bacteria also include the intrinsically acid-tolerant genera such as *Lactobacillus* [23] and *Bifidobacterium* [37]. Although *S. mutans* and *Streptococcus sobrinus* were the first acid-tolerant streptococcal species identified, today it is known that other streptococci such as *S. oralis, Streptococcus mitis*, *S. sanguinis* and *Streptococcus anginosus* also can exhibit acid-tolerance and indeed *S. oralis* can produce acid at rates exceeding that of some *S. mutans* strains [7, 20, 30, 38]. In this study, images of plaque biofilms from individuals with a high acid-tolerance score revealed a range of bacterial morphologies amongst the acid-tolerant cells, indicating that a variety of bacterial species probably contribute to the overall acid-tolerant microbiota of the biofilm.

Irrespective of the specific bacterial composition, a high degree of acid tolerance would enhance the cariogenic potential of the supragingival biofilm and the appearance of an acid-tolerant microbiota would be expected to be an early event in the caries process. Thus, it may be possible to utilize evaluation of the overall level of acid tolerance in oral biofilms as an early marker to predict enamel caries. In this study, the proportion of acid-tolerant microorganisms in biofilms collected from the study population ranged from low to high on the scale (Fig. 2), confirming that the method has the capacity to distinguish between individuals with different levels of biofilm acid-tolerance. The majority (92%) of the subjects showed low levels (scores 1–3) suggesting that they lack the pre-requisite conditions necessary for development of caries and would be expected to have a low risk for disease development. This figure corresponds well to the proportion of caries-free 12-year olds in the Health Authority area [39]. On the other hand, 8% had a high level of acid-tolerant bacteria (score 4 or 5), suggesting that these individuals may have an increased risk of developing caries.

The oral cavity is known to contain a number of distinct ecological niches influenced by factors such as nutrient supply, pH and oxygen tension. For instance, the properties of subgingival biofilms are known to differ from those of biofilms above the gingival margin or on the tongue. To investigate whether there are differences between supragingival sites within subjects, acid tolerance in approximal plaque from the incisor, pre-molar and molar regions was compared. The acid-tolerance was shown to be largely consistent for the three selected sites within an individual; with complete agreement between the values for the incisor and molar regions. This suggests that supragingival biofilms on different teeth are subjected to the same stress factors and ecological influences of relevance for acid-tolerance development [1].

To investigate the reliability of the assessment method for acid-tolerance of the biofilms, two samples were taken from the same site 3 days apart. In 19 of 20 individuals, biofilm acid tolerance was shown to be low at baseline and the same outcome was seen after 3 days. This confirms that the method is reliable since detectable changes in acid-tolerance are unlikely to occur during this time. Score 4 and 5 represents a dysbiotic state indicating high carbohydrate intake and low pH over time. A pre-requisite for the development of an acid tolerant microbiota is acid-adaptation and the enrichment of aciduric strains within the biofilm [5] and since bacterial doubling time in dental biofilms in vivo is known to be in excess of 21 h, it is unlikely that acid-tolerant bacteria would achieve numerical dominance in the biofilm over 3 days. It is therefore also unlikely that any food intake prior to sampling would affect the result. However, to investigate more closely how rapidly changes in biofilm acid tolerance can occur, samples were retaken from the same site after 1 month. At 51 of 60 sites, the acid-tolerance at baseline was low or moderate and no changes were seen between samples taken at baseline and at 1 month. However, the acid tolerance increased at 4 and decreased at 5 sites over the 1-month period suggesting that both acid-adaptation as well as de-adaptation can occur over this time. Acid adaptation has previously been demonstrated to occur over 10 days in in vitro experiments in chemostats using a 9-species-consortium that was pulsed daily with glucose [40].

At present there are no marker-based methods to identify acid tolerant bacteria. However, exposing bacteria to low pH is commonly used to distinguish acid tolerant from non-acid tolerant ones. This method is based on the ATR concept – where an exposure to sub-lethal pH values (pH 5.5) leads to an ATR that enhances survival at pH values which kill non-adapted cells (killing pH) [9]. Bacteria that have been exposed to sub-lethal pH in the oral cavity due to metabolism of carbohydrates would be acid-adapted and thus when exposed to a killing pH (pH 3.5) ex vivo these adapted bacteria as well as intrinsically acid tolerant ones will survive whereas not acid tolerant will not. The killing pH was chosen based on previous studies on acid tolerance capacity of different oral species [7]. Acid tolerant bacteria (defined as viable cells after the ex vivo pH challenge) were distinguished from non-acid tolerant ones using the LIVE/DEAD® BacLight™ Viability stain. The LIVE/DEAD stain have been tested on a range of different Gram positive and Gram negative species and have shown good correlation with standard plate counts [41].

In order for enamel caries lesions to develop and progress, extended periods of low pH within biofilms are required. Studies have shown that during cariogenic challenge caries lesions can develop within 14 days although under normal circumstances caries development and progression is a much slower process [42–44]. Therefore, acid-tolerance in biofilms within the subjects over 1 year was also investigated. As seen in the short-term investigation, in the majority of individuals the acid-tolerance at baseline was low and this was maintained at 6 months and 1 year. However, in a minority of subjects a major increase (4 scores) was seen between the level of acid tolerance and baseline and that at 6 months. In two individuals, the reverse appeared to have occurred where high acid tolerance levels at baseline were found to be low at both 6 months and 1 year. Thus succession towards a healthier microbiota appears to have occurred in these individuals, suggesting that the ecological pressure driving development of acid tolerance has been removed in these subjects. In one individual, a low acid tolerance score at baseline had increased to a high score at 6 months, which was also seen at 1 year. This suggests that the normal homeostasis within the biofilm in this subject was disrupted leading to extended periods of low pH and succession of acid-tolerant bacteria. Factors potentially driving this process could be frequent intake of fermentable carbohydrates or reduced buffer capacity in saliva.

## Conclusion

Biofilm acid tolerance showed short-term stability and low variation between different sites in the same individual. The acid tolerance test is an indicator of the acid-producing potential of a biofilm and is thus intimately related to the demineralisation process. As such it represents a promising biological biomarker candidate for caries prediction. However further evaluation of the test is needed and prospective clinical trials are called for to evaluate the diagnostic accuracy.
